# A Review of Alpha-Gal Syndrome for the Infectious Diseases Practitioner

**DOI:** 10.1093/ofid/ofaf430

**Published:** 2025-07-21

**Authors:** Akira A Shishido, Gary P Wormser

**Affiliations:** Division of Infectious Diseases, Virginia Commonwealth University, Richmond, Virginia, USA; Division of Infectious Diseases, New York Medical College, Valhalla, New York, USA

**Keywords:** alpha-gal syndrome, *Amblyomma americanum*, lone star ticks, red meat allergy

## Abstract

Alpha-gal syndrome (AGS) is an emerging allergic disease caused by an immunoglobulin E (IgE) response to galactose-α-1,3-galactose (alpha-gal), a sugar found in mammalian meat. The initial IgE sensitization follows a bite from the *Amblyomma americanum* (lone star) tick in the United States, although other tick species can also cause the disease. AGS presents with delayed symptoms, primarily gastrointestinal or allergic reactions like urticaria or anaphylaxis, hours after mammalian meat consumption. The nonspecificity of the symptoms can delay the diagnosis of AGS for years. Diagnosis relies on clinical evaluation and detection of alpha-gal–specific IgE in blood. As the lone star tick's range is expanding beyond the southern United States, AGS is gaining recognition in other regions. However, many healthcare providers remain unaware of the condition, leading to misdiagnosis. Infectious diseases physicians who frequently evaluate patients with tick exposure must be familiar with AGS, including tick identification, bite prevention, and knowledge of the alpha-gal–containing vaccines.

## Teaser

Alpha-Gal Syndrome is an emerging allergic disease caused by tick bites, about which many providers remain unaware. In a narrative review, we summarize the critical information known about the disease that is relevant to infectious disease providers.

Alpha-gal syndrome (AGS), also known as “red meat allergy,” is a condition where individuals experience allergic or gastrointestinal symptoms after eating mammalian meat, typically with a delayed onset [1]. Alpha-gal (AG) refers to the oligosaccharide galactose-alpha-1,3-galactose that is present in nonprimate mammals [2–4]. The human B-group blood antigen is nearly identical to AG but has an additional fucose on its penultimate galactose residue [3]. Humans and nonhuman primates do not express AG and develop immunoglobulin G (IgG), immunoglobulin M (IgM), and immunoglobulin A (IgA) serum antibodies against it without adverse effects [3]. Only the presence of immunoglobulin E (IgE) antibodies to AG causes AGS [5, 6]. The primary cause of IgE sensitization to AG in the United States (US) is the bite of *Amblyomma americanum* ticks, also referred to as lone star ticks (LSTs) [7, 8]. However, AGS is recognized worldwide, with multiple other tick species implicated outside of the US [9–18].

Many clinicians remain unaware of this illness. A 2022 survey of 1500 general practitioners revealed that 42% were not aware of AGS, and among those who were aware, 35% lacked confidence in their ability to diagnose and manage AGS [19]. One study found that most patients first propose the diagnosis themselves, without input from a healthcare provider [20]. Some patients may wait over 10 years before they are diagnosed and may undergo unnecessary procedures such as radiologic imaging and endoscopies [21, 22]. The Centers for Disease Control and Prevention estimates that up to 400 000 cases of AGS have occurred in the US since 2010 [23]. Importantly, AGS can affect any age group, and many pediatric cases have been reported, although AGS appears to be less common in children than in adults [24, 25].

As AGS cases increase, adult and pediatric infectious diseases (ID) practitioners must be able to recognize this emerging disease and understand those aspects most relevant to them [19, 23, 26]. Herein, we review the current understanding of AGS, discuss open questions, and provide certain information relevant to ID practitioners.

## BACKGROUND

### The Alpha-Gal Syndrome

AGS can present with symptoms of a severe food allergy that may include angioedema, urticaria, and anaphylaxis [27]. However, unlike most other food allergies, these symptoms occur in only a minority of patients with AGS [27]. Milder AGS presentations include gastrointestinal symptoms like abdominal pain, pyrosis, nausea, vomiting, and diarrhea, as well as other symptoms such as arthralgia and pruritis [27]. Unexpectedly, having gastrointestinal symptoms appears to be the most common clinical presentation [27, 28]. Consequently, many patients are initially incorrectly diagnosed with irritable bowel syndrome [27, 29]. Unlike other food allergies, AGS symptoms usually appear with a delayed onset of 2–6 hours after consuming mammalian meat [27, 30]. Indeed, many symptoms first occur after 10 Pm and consequently may be unknowingly attenuated by patients who take diphenhydramine as a sleep aid [27]. Symptom onset can be accelerated by consuming a large amount of meat and by factors such as alcohol, nonsteroidal anti-inflammatory drugs, increased activity, stress, lack of sleep, and menses, which might affect intestinal permeability and allow quicker AG release into the bloodstream [27, 31–33].

Symptoms related to the development of AGS typically first occur 1–4 months after a LST bite [22, 27, 34]. However, the timing between a recognized tick bite and the onset of symptoms of AGS can range from a few weeks to several years [8]. In those who do recall a recent tick bite, they often describe the bite site as unusually erythematous and pruritic compared with earlier tick bites [22, 27]. The proportion of AGS patients who recall a tick bite is variable across studies and likely affected by recall bias; however, it is estimated that up to 50% of cases may not remember being bitten by a tick [8]. It is not presently known how long the tick must remain attached to induce AG sensitization; however, animal models suggest that a few days are needed to induce the production of AG IgE [35, 36].

### The Current Model of Understanding of AGS and the Association With Tick Bites

Allergic reactions mediated by IgE antibodies to AG were first described in 2007 in a subset of cancer patients who had been treated with the monoclonal antibody cetuximab [1, 37]. O’Neil et al reported that 22% of patients in North Carolina and Tennessee who received cetuximab developed severe anaphylactic or urticarial reactions, while <3% of patients receiving cetuximab nationally experienced these reactions [37]. Chung et al then performed an analysis on 25 patients from the southern US who had a hypersensitivity reaction to cetuximab and found that 17 (68%) had preexisting IgE antibody specific to cetuximab [38]. This finding contrasted with a comparison group of control subjects from Boston, Massachusetts, from which only 2 of 341 (0.6%) subjects treated with cetuximab had IgE antibody directed to the drug [38]. Eventually, the target of the cetuximab-specific IgE antibody was confirmed to be AG, which is present on the Fab portion of the cetuximab heavy chain [38, 39].

In 2009, Commins et al found that 24 patients with new allergic reactions to red meat also had IgE antibody specific for AG [40]. Commins et al later noted that the geographic distributions of patients with red meat allergies and those who experienced severe cetuximab reactions resembled that of the tick-transmitted infections Rocky Mountain spotted fever and human monocytic ehrlichiosis [1]. They postulated that IgE antibodies to AG may have developed from bites by the LST [1]. The authors found that AG IgE and total IgE increased by at least 20-fold after a tick bite by comparing these test results on serum obtained before and after tick bites in 3 patients [1]. Two of the 3 patients went on to develop episodes of urticaria starting 3–4 hours after eating red meat [1]. Several subsequent meat challenge studies also supported the link between IgE to AG and an allergic reaction to red meat [41, 42]. Mitchell et al further supported the link between tick bites and AG IgE with a prospective study of outdoor workers that demonstrated an average increase in AG IgE levels of 1.99 IU/mL following 1 or more tick bites [43].

While additional case reports reinforce the link between tick bites and AGS, none included laboratory testing for IgE to AG both pre– and post–tick bite exposure, as was done by Commins et al in 2011 [1, 22, 44, 45]. Kersh et al conducted a case-control study of 82 AGS patients and 191 controls in a region endemic for LSTs [46]. They found that patients with AGS were significantly more likely to report 4 or more tick bites and noted localized reactions to tick bites significantly more often than controls without AGS [46].

Outside of these studies, evidence supporting the link between AGS and tick bites in the US is retrospective, indirect, or based on animal model data. Multiple studies demonstrate that LSTs possess AG moieties in their saliva [47, 48]. Studies with AG-deficient mice immunized with LST tick salivary gland extract, or bitten by LSTs, showed the development of AG IgE and subsequent anaphylactic responses to red meat [35, 49–51]. A systematic review in 2021 concluded that the available evidence suggests that tick bites may lead to AG IgE development, but with AGS only occurring in a subset of those with IgE to AG [8]. However, additional studies should further clarify the disease process, risk factors, and sequelae [8].

### Risk Factors

As mentioned above, AG and the B-group blood antigen differ in structure by only a single fucose, potentially resulting in antibody cross-reactivity [2, 3, 52, 53]. The B-group antigen affords protection against developing AGS, although protection is not 100% [24, 52, 54]. In contrast, a history of atopy may increase the risk of AGS development [12, 55, 56].

AG IgE has also been linked to cat ownership [55] and to cat flea bites [57]. Trombiculidae larvae (chigger) bites have also been linked to AGS [58]. Patients with AGS also appear to more likely have elevated rates of sensitization to insect venom (bee, wasp, hornet, or the fire ant), are more often male, and more often live in rural areas and have outdoor lifestyles; however, these factors may just be risk modifiers for increased tick exposure [12, 24, 46, 59–61].

The most compelling non–tick bite factor that may contribute to AGS development is exposure to the helminth *Ascaris lumbricoides* [18]. Murangi et al found that high *Ascaris* IgE levels correlated more strongly with AGS than did tick bites in Africa [18].

A recent series of AGS cases in the midwestern US, including some areas in which LSTs were not previously known to be present, highlights the question of whether there are additional clinically significant causes of AG sensitization that have not been defined [23, 62, 63].

### Open Questions

Many aspects of the pathophysiology of AGS remain incompletely understood (Table 1). Most individuals with IgE to AG do not develop AGS, and it is not clear what additional factors dictate symptom development [12, 56, 60, 64–66]. Human exposure to AG in tick saliva specifically leads to the production of IgE antibodies to AG, while exposure to AG in the gastrointestinal tract results only in IgM, IgG, and/or IgA antibody production. The difference in these findings and how tick bites specifically drive an IgE response is not entirely clear but is highly pertinent to the development of AGS [5, 48, 67–70]. Of note, while allergy to protein antigens is common, carbohydrate-induced allergies are rare [71]. AGS symptoms can also depart from classic allergic and anaphylactic reactions; gastrointestinal manifestations appear to be most common, while itching of the tongue and throat occurs in <1% of cases [27]. The explanation for this heterogeneity in symptoms is poorly understood [3, 7].

**Table 1.** Open Questions Regarding Alpha-Gal Syndrome Pathophysiology and Proposed Studies to Answer These Questions

Open Questions	Potential Studies
Why do AGS symptoms differ from classic anaphylactic food reactions?	Food challenge studies with cytokine profiling or transcriptome analysis and mast cell and/or basophil localization
Why are AGS symptoms so delayed after triggering exposures (2–6 h)?	In vitro exposure of primed mast cells to AG glycolipids; food challenge studies with cytokine profiling or transcriptome analysis
What causes the different phenotypes of disease (ie, gastrointestinal vs systemic allergic symptoms)?	Challenge studies that include serial measurements of AG IgE levels and immune profiling
Why are *Ixodes scapularis* ticks not associated with AGS?	Tick feeding and saliva analysis for various tick stages and feeding times; pre– and post–tick bite serologic testing for IgE to AG
Why don't tick bites per se trigger AGS symptoms?	Mouse-model challenge studies with cytokine profiling or transcriptome analysis; human challenge studies
How and why do LSTs drive specifically an IgE response to AG?	In vivo sensitization of AG knockout mice and measurement of IgE vs other immunoglobulins, cytokine profiling, and basophil activation; tick saliva analysis of immunologic factors that potentially drive IgE development in humans who are bitten
Does AG sensitization increase the risk of coronary artery atherosclerosis? What percentage of AG sensitized patients have CAD because of it? Does dietary modification reduce this risk? Does tick bite avoidance reduce this risk? Can it be the only manifestation of AGS?	Case-control studies in larger populations Large cohort analysis with varying dietary habits Analysis of CAD in AGS patients vs controls followed over time
Are there other causes of AG sensitization besides the LST in the United States?	Population studies in regions without LSTs
Why do only some patients with AG IgE develop AGS, while many others do not?	Large cohort and case-control studies evaluating clinical symptoms, IgE levels to AG, and other potential modifying factors
Does AG IgE sensitization contribute to arthritis/arthralgia? What is the pathophysiology behind this?	Cohort and case-control studies focusing on musculoskeletal symptoms

Abbreviations: AG, alpha-gal (galactose-α-1,3-galactose); AGS, alpha-gal syndrome; CAD, coronary artery disease; IgE, immunoglobulin E; LST, lone star tick.

Additionally, it is unclear why bites from only certain AG-expressing tick species are associated with AGS in the US [72]. Like LSTs, *Ixodes scapularis* ticks also possess AG in their saliva [47]. Additionally, *I scapularis* salivary samples stimulated basophils primed with plasma from AGS subjects in vitro [47]. However, *I scapularis* tick bites have historically not been associated with AGS development [47, 72, 73]. Variations in tick saliva composition, feeding behavior, and other ecologic factors for *I scapularis* ticks have been suggested as explanations, but remain unproven [47, 72]. Other *Ixodes* species ticks (for example, *Ixodes ricinus*), however, have been linked to AGS in Europe [74]. In addition, 2 recent case reports in the US linked the development of AGS to *Ixodes* tick bites (1 to an *I scapularis* bite and 1 to an *Ixodes pacificus* bite), suggesting that this potential connection should be further investigated [75–77].

Interestingly, after sensitization, tick bites per se do not trigger AGS symptoms, whereas red meat ingestion frequently does [27, 71, 78]. AGS improves over time with tick avoidance, while most other food allergies in adults do not improve over time [27, 71]. AGS reactions are delayed, starting several hours after meat ingestion, whereas for most other food allergies, symptoms present rapidly after consumption of the antigen [31, 71]. The leading theory explaining the symptom delay attributes it to the time required for AG-containing glycolipids to exit the gastrointestinal tract and enter the bloodstream via the thoracic duct [3, 7, 48, 79–81]. This theory proposes that allergic symptoms do not occur until these glycolipids interface with tissue mast cells [3, 7, 79–81]. This model, however, does not address why gastrointestinal symptoms would also be delayed.

Of note, AG sensitization has been linked to coronary artery disease (CAD) [7, 82–85]. In a study of 1056 patients undergoing coronary angiography and 100 with ST elevation myocardial infarction (STEMI) in Australia (where *Ixodes holocyclus* ticks cause AGS), Vernon et al found that AG sensitization (AG IgE ≥0.10 IU/mL) was significantly linked to noncalcified plaques and obstructive CAD, independent of age, sex, and traditional risk factors [84]. Additionally, AG sensitization was 12.8-fold higher in patients with STEMI compared to matched controls [84]. While this association requires further study, existing literature has linked mast cells and total serum IgE with atherosclerosis, making this a plausible association [7, 86, 87]. Given the undesirable implications of CAD development, further studies should be done to explore this connection, and if confirmed, to assess what modifications (ie, diet modification, tick avoidance) might favorably impact this risk. For example, and of major relevance, if avoiding red meat is unnecessary for a particular patient to prevent AGS symptoms, should it still be done to prevent CAD?

Recent data have also linked AG IgE to the presence of certain chronic musculoskeletal symptoms. For example, a study performed in a population in which 17.5% had an AG IgE level of >0.1 IU/mL found that AG sensitization was associated with knee pain, aching, or stiffness, while antibodies to *Ehrlichia* and *Rickettsia* were not [88]. Further studies, however, are needed to validate this association.

Like many immunologic diseases, AGS is based on a multifactorial process that includes host predisposition and environmental exposures. LST bites appear to be the primary cause of AGS in the US, although many questions about other potential exposures remain unanswered.

## CLINICAL CONSIDERATIONS FOR THE ID PRACTITIONER

AGS does not appear to be a sequala of a tick-borne infection per se, only of a tick bite. For example, there is no evidence suggesting a correlation between IgG antibodies to either *Ehrlichia* or *Rickettsia* and AGS [89]. Interestingly, like many microorganisms, *Borrelia burgdorferi*, the spirochete responsible for causing Lyme disease, expresses AG [33]. However, in the US, *B burgdorferi* is principally transmitted by *I scapularis* ticks, bites from which historically have not been associated with AGS.

In addition to *B burgdorferi*, *Anaplasma phagocytophilum*, *Mycobacterium* spp, *Plasmodium* parasites, *Trypanosoma*, and *Leishmania* also express AG [6, 33, 90–92]. Based on observational and animal model data, Cabezas-Cruz et al hypothesized that AGS patients who are blood group B negative may produce high levels of AG IgG and IgM that at least partially may protect these individuals from infections caused by such AG-expressing pathogens [6, 33, 90–92]. In addition, the authors also postulated that the development of AG IgE antibodies enables humans to develop tick bite resistance, a phenomenon in which IgE-primed basophils cluster around tick mouth parts embedded in the skin, potentially producing a protective response [93].

There are several scenarios in which the ID provider may interface with patients with AGS or potentially at risk for AGS (Table 2). The most common scenario is when a patient presents after a tick bite with either no symptoms or with nonspecific symptoms. ID providers need to understand regional tick presence and the associated disease risks, identify the tick that has bitten a patient if feasible, provide counseling on preventing future tick bites, and make appropriate referrals if AGS is a consideration.

**Table 2.** Clinical Scenarios in Which Infectious Diseases Practitioners May Be Involved With Alpha-Gal Syndrome

Scenario	Pertinent Information
Presentation after tick exposure with no symptoms	Did a tick bite occur? Attachment time? Tick identification, consider: Geographic region in which tick bite was acquired Location of bite on the body Seasonality Size and appearance of tick; Is AG testing indicated? Consider risk for TBIDs
Referral to ID after tick exposure with nonspecific and/or unexplained symptoms	Are symptoms compatible with AGS? Or with a TBID? Is AG testing indicated?
Referral to ID for treatment of TBID	Confirm and treat TBID Is AG testing indicated? Counsel patient on possibility of sensitization to AG, implications of positive AG IgE, development of AGS, and potential association with cardiovascular disease; consider referral to an allergist
Established diagnosis of AGS with questions about safety of certain vaccines	Overall risk of reaction to vaccines is low For vaccines with a potential risk, consider alternative vaccine options if available (Table 3) Counsel on general risks associated with certain medications (heparin, mAbs, gelatin, magnesium stearate, glycerin, antitoxins)

Abbreviations: AG, alpha-gal (galactose-α-1,3-galactose); AGS, alpha-gal syndrome; ID, infectious diseases; IgE, immunoglobulin E; mAbs, monoclonal antibodies; TBID, tick-borne infectious disease.

### *Amblyomma americanum* (Lone Star) Tick Characteristics and Identification

**Figure 1.**
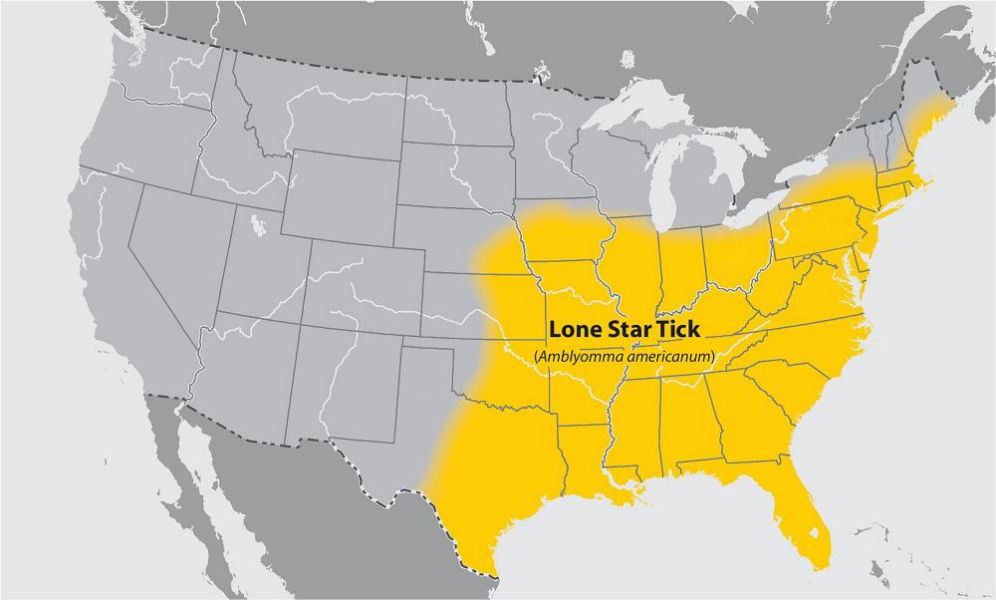
Geographic range of *Amblyomma americanum* lone star ticks, 2011. Image courtesy of the Centers for Disease Control and Prevention [96].

The LST is an aggressive tick prevalent in the eastern, southeastern, and south-central US with an actively expanding geographic range (Figure 1) [26, 94–96]. LSTs are found in woodland habitats, particularly in forests with dense underbrush [97, 98]. Unlike *I scapularis* and *Dermacentor variabilis* ticks, LSTs are often found in manicured habitats, including turf lawn, picnic areas, and even on paved pathways [99].

**Figure 2.**
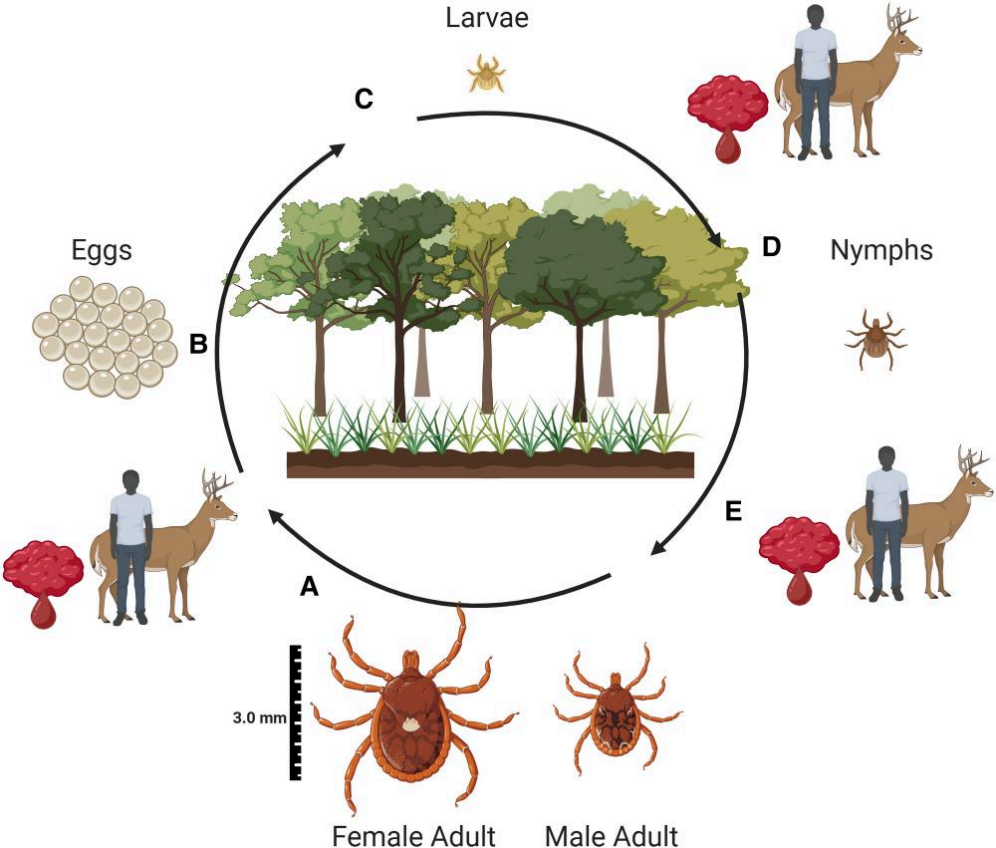
The life cycle of the lone star tick. *A*: The adult female tick takes a blood meal from host (white-tailed deer and human depicted). *B*: After several days, the female drops from the host and lays up to 5000 eggs, typically in a humid area at soil level [100]. *C*: The eggs then hatch into larvae, which after a quiescent period, quest for a host. *D*: The larva takes a blood meal lasting 1–3 days, then drops from the host to molt into a nymph [100]. *E*: Nymphs repeat this process, and after a blood meal molt into adults. The entire life cycle takes approximately 2 years in nature [100]. Figure created with BioRender.com.

The LST life cycle involves 3 stages: the adult, nymphal, and larval stages, as described in Figure 2. LSTs most commonly bite the groin, pelvis, and thighs, followed by the upper extremities [101]. This bite distribution differs from *I scapularis* ticks, which distribute bites more evenly over the body [101].

**Figure 3.**
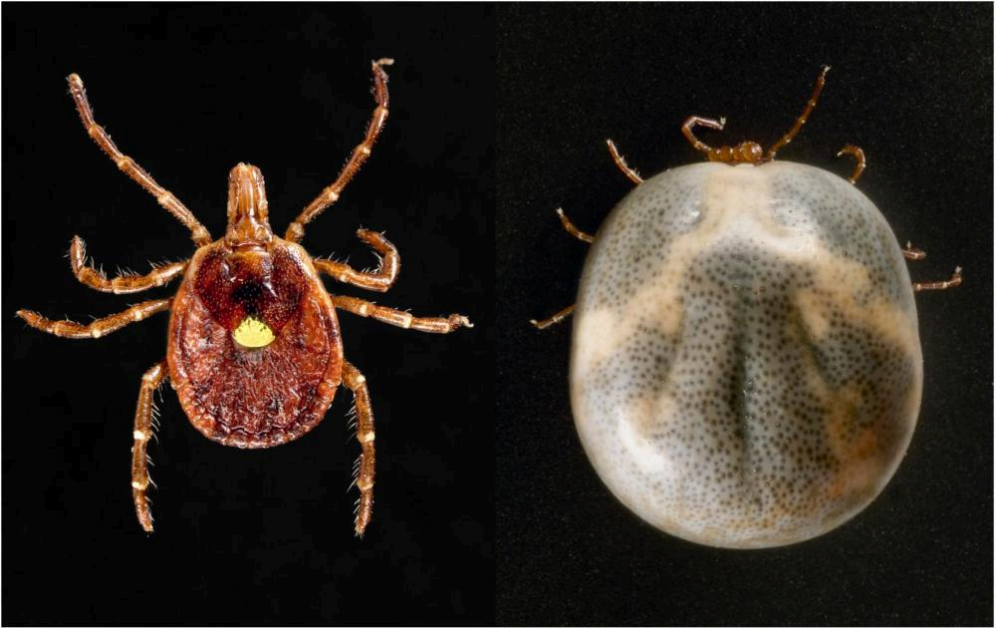
Adult female lone star tick (left) and engorged after a blood meal (right). Images courtesy of the Centers for Disease Control and Prevention Public Health Image Library [102, 103].

**Figure 4.**
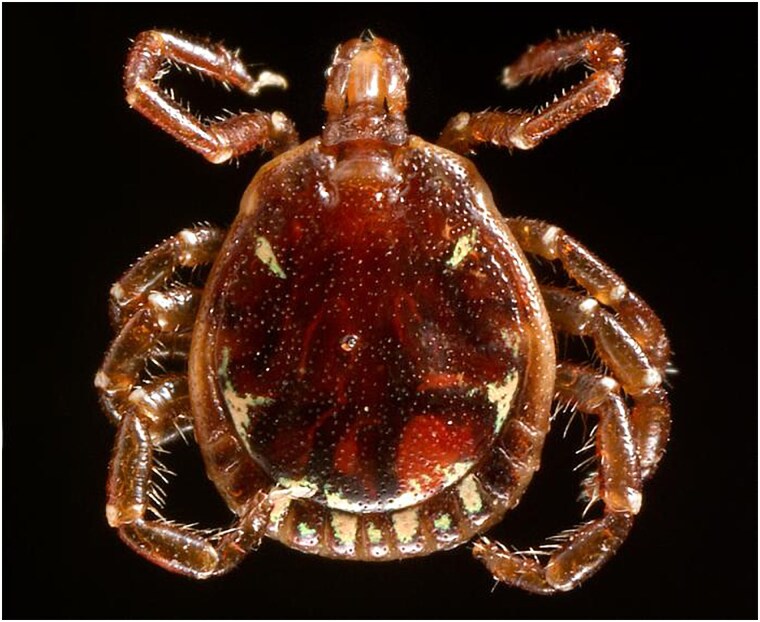
Adult male lone star tick. Image courtesy of the Centers for Disease Control and Prevention Public Health Image Library [104].

LSTs are larger than *I scapularis* ticks. Adult female LSTs are the largest tick stage, extending beyond 3 mm in length, and recognized by the gold “star” on the center of the back (Figure 3) [100]. Adult males have white streaks or spots around the margins of their body (Figure 4) [100]. Larvae and nymphs are challenging to identify due to their smaller size (0.5–1.0 mm in length) [100]. Adult LST populations peak from April to June, whereas larval and nymphal stages are mainly present from July to September [100].

LST bites can transmit infectious agents to humans, with *Ehrlichia chaffeensis*, the cause of human monocytic ehrlichiosis, being the most common; these infections are discussed elsewhere [99, 100, 104, 105].

### Diagnosis of AGS

**Figure 5.**
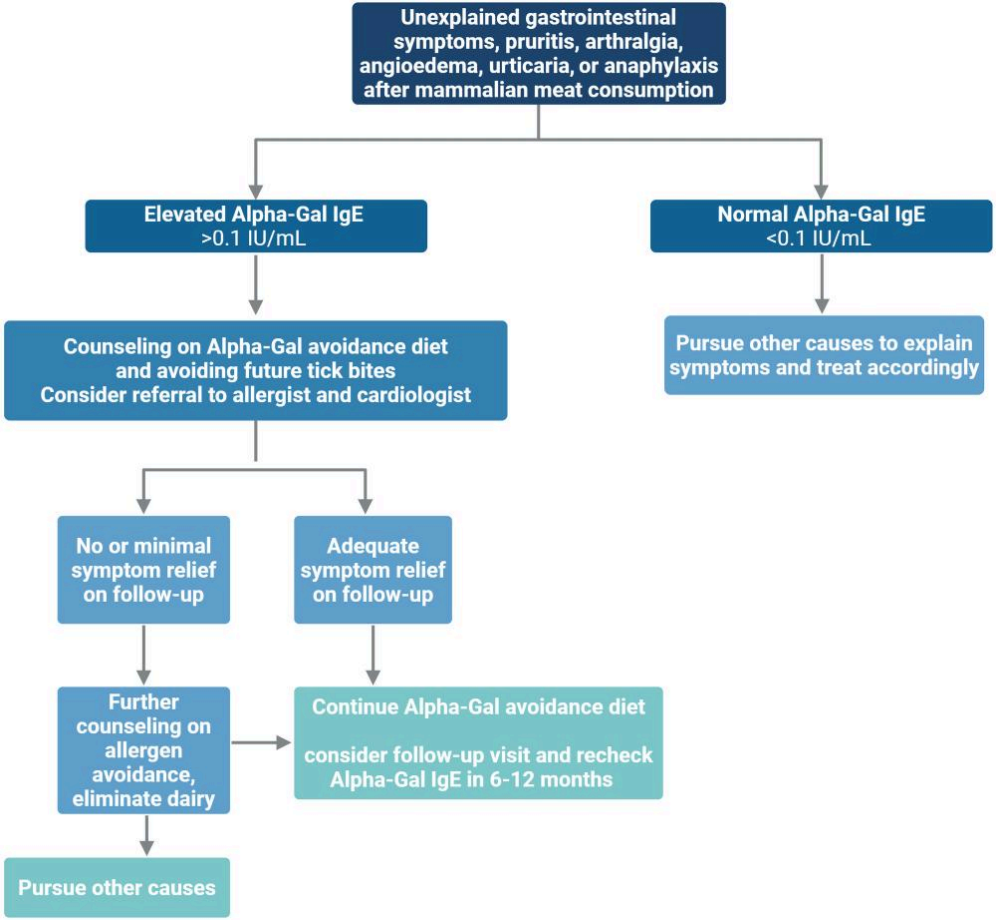
Diagnosis and management of alpha-gal syndrome. Modified and updated from Lee et al [29] and McGill et al [28]. Created with BioRender.com. Abbreviation: IgE, immunoglobulin E.

Diagnosis of AGS is based on a compatible clinical history with evidence of AG sensitization (Figure 5). There are no clinical guidelines establishing the titer of AG IgE needed to confirm the diagnosis. However, most reports use the cut-off of >0.1 IU/mL as a positive result [24, 27]. The Council of State and Territorial Epidemiologists has established a surveillance case definition for AGS that includes this value [42, 106]. This result has a reported sensitivity of 100% [107]. However, a higher value of ≥2 IU/mL, or a value >2% of the total IgE antibody level, may be more specific, given the high background seroprevalence rates in certain regions that can exceed 30% [64, 108]. Skin-prick testing has also been used to diagnose AGS, but is more difficult to interpret, and therefore not as widely used as AG IgE antibody testing [27, 106, 107, 109]. The titer of AG IgE in blood does not appear to predict AGS symptom severity [27, 31]. There is some evidence that a basophil activation test may differentiate AGS patients from those who are sensitized without symptoms [110].

### Considerations for the Asymptomatic Tick-Bitten Patient

When a patient presents after a LST tick bite and is asymptomatic, should he or she be screened for AG sensitization? It is estimated that baseline AG sensitization in an endemic region can be greater than 30% [64] and that as few as 4.8%–8.6% of individuals with detectable AG IgE levels will develop clinical AGS [23, 28, 46]. However, if the recent evidence suggesting that asymptomatic patients with AG IgE may be at risk for CAD is confirmed in future studies, screening an asymptomatic patient presenting at least 4 weeks after a confirmed LST bite for AG IgE may be reasonable. However, it is not yet clear whether dietary modifications or tick bite avoidance would modify the CAD risk [82–85].

### Management of AGS

Allergen avoidance is the cornerstone of management [27, 28, 108]. Patients should avoid beef, pork, venison, and other mammalian meat products [27, 28, 108]. Dairy products also contain AG, but typically can be tolerated [27, 28, 108]. If patients do not have systemic allergic symptoms, it is reasonable to recommend an AG avoidance diet but still permit dairy. If patients have ongoing symptoms on an AG avoidance diet, then they should avoid dairy products as well [27, 108].

Porcine- or bovine-based products such as gelatin, marshmallows, gummy bears, and other desserts may not be tolerated [27, 28, 108]. Fish, seafood, and poultry, however, can always be eaten safely [27, 28, 108]. AGS reactions from inhalation of aerosolized AG created by frying bacon or beef products have been reported [28].

Caution is also advised for dining out, as restaurants may add lard or suet to fryers (ie, French fries) and unintentionally contaminate foods [27, 28, 108]. Labels that state “natural flavoring” may mean pork or beef ingredients [27]. In addition, some toothpastes and skin creams (containing lanolin) may also need to be avoided [27].

Eating mammalian meat does not appear to trigger an IgE response to AG. However, repeated tick bites can increase AG IgE blood levels and avoiding them can reduce AG IgE antibody levels [1, 27, 43, 46, 111]. Repeat tick bites will not trigger AGS reactions, a surprising observation that is of interest and needs to be better understood [1, 7, 27, 108]. AGS patients should make efforts to avoid future tick bites. ID and other providers should advise AGS patients to perform daily total body tick checks, shower or bathe after outdoor activity, treat clothes and footwear with permethrin, and apply insect repellents containing N,N-Diethyl-Meta-Toluamide, picaridin, IR3535, or oil of lemon eucalyptus to exposed skin [112, 113] (see https://www.cdc.gov/ticks/prevention/index.html). AGS patients with pets that go outdoors should regularly brush them and administer standard veterinary flea and tick preventive medications. As mentioned earlier, bee, wasp, and hornet stings may also lead to increased AG IgE and should be avoided.

Certain drugs and other medical exposures are also relevant to patients with AGS. Cetuximab, abatacept, and infliximab each contain AG [27, 108]. Heparin is derived from porcine or bovine sources and may cause AGS reactions [27, 108]. However, the doses used for deep venous thrombosis prophylaxis are usually tolerated [27]. Larger boluses of heparin used in heart catheterization, valve procedures, or extracorporeal membrane oxygenation are more likely to trigger reactions [27, 108]. Premedication with antihistamines or steroids can be considered, and in rare cases, an alternative anticoagulant may be needed [27, 108]. Pancreatic enzymes, thyroid hormone, bioprosthetic heart valves, and gelatin-containing medications have also been reported to trigger AGS [27, 108].

It is particularly important for the ID practitioner to be aware of the vaccines that contain gelatin, which include live intranasal influenza, measles-mumps-rubella, varicella, oral typhoid, rabies, and yellow fever vaccines (Table 3). While an analysis of 2684 patients sensitized to AG and 25 patients with AGS found no significantly increased risk of anaphylaxis after receiving vaccines, with and without gelatin, we recommend considering gelatin-free alternatives or assessing safety of these products by skin-prick testing performed by an allergist before administering these vaccines [114, 117]. The injectable influenza vaccine Flucelvax is grown in canine kidney cells, thus posing a theoretical risk [115]. Antivenoms and other immunoglobulin-based products derived from horses or sheep may contain AG [27, 108, 118]. To our knowledge, no antibiotics, antivirals, or antifungal agents contain AG; however, some formulations may come in gelatin capsules, or contain magnesium stearate or glycerin from mammalian sources [118].

**Table 3.** Alpha-Gal–Containing Vaccines and Proposed Alternatives

Vaccine (Name, Manufacturer)	Formulation	Gelatin Content (per Stated Dosage)	Gelatin-Free Alternative (Name, Manufacturer)
Influenza (FluMist, MedImmune)	Nasal inhalation	2 mg per 0.2 mL	Injectable flu vaccines other than Flucelvax
Influenza (Flucelvax^a^, Seqirus)	IM injection	0^a^	Injectable flu vaccines other than Flucelvax
Measles, mumps, rubella (MMRII, Merck)	IM or SC injection	14.5 mg per 0.5 mL	PRIORIX, GlaxoSmithKline
Measles, mumps, rubella, varicella (ProQuad, Merck)	IM or SC injection	11 mg per 0.5 mL	PRIORIX + VARILRIX^b^, GlaxoSmithKline
Rabies (RabAvert, Novartis)	IM injection	12 mg per 1.0 mL	Imovax rabies, Sanofi Pasteur
Typhoid Vaccine Live Oral Ty21a (VIVOTIF, Crucell)	Oral capsule	Capsule	Typhim Vi, Sanofi Pasteur
Varicella (VARIVAX, Merck)	IM or SC injection	12.5 mg per 0.5 mL	VARILRIX^b^, GlaxoSmithKline
Yellow fever (YF-VAX, Sanofi Pasteur)	SC injection	7.5 mg per 0.5 mL	STAMARIL^c^, Sanofi Pasteur
Zoster (ZOSTAVAX, Merck)	SC injection	15.58 mg per 0.65 mL	SHINGRIX, GlaxoSmithKline

Modified and updated as of April 2025 from Kelso et al [114]. When no alternative vaccine is available, skin-prick testing should be performed by an allergist prior to challenging with these vaccines.

Abbreviations: IM, intramuscular; SC, subcutaneous.

^a^Flucelvax, made by Seqirus, Inc, does not contain gelatin but is propagated in canine kidney cells, which poses a theoretical risk for patients with alpha-gal syndrome and therefore is not recommended (Scott Commins, MD, personal communication) [115].

^b^VARILRIX is not licensed in the United States [116].

^c^STAMARIL is not licensed in the United States [116].

In instances of persistent symptoms despite strict dietary avoidance of AG, adjunctive medical therapies can be considered. Expert opinion suggests that long-acting antihistamines or use of oral corticosteroids are beneficial [27]. Oral cromolyn solution can be used to reduce gastrointestinal symptoms [27]. Any patients with systemic or anaphylactic symptoms (eg, face or throat swelling, voice changes, breathing difficulty, hives, or fainting) should be provided an epinephrine auto-injector and referred to an allergist [28].

Over time, AG IgE levels will decrease if patients avoid tick bites, but the rate of decline varies [17, 108, 119]. After 6–12 months of allergen avoidance and resolution of symptoms, it is reasonable to recheck AG IgE levels [120, 121]. If levels decrease to <0.1 IU/mL, patients may tolerate mammalian meat products again; however, any food challenges should be supervised by an allergist experienced with this condition [27, 28, 108, 121].

## CONCLUSIONS

Cases of AGS continue to rise in the US following the expanding geographic range of LSTs [23, 26]. As symptoms of AGS are protean and differ from other food allergies, they may be incorrectly attributed to other causes, including infections; therefore, ID practitioners should possess a working knowledge of AGS, since the primary cause of AGS in the US is a LST bite. Patients with AGS are often incorrectly diagnosed for many years, as classic anaphylactic presentations are less common than delayed onset of nonspecific gastrointestinal symptoms. Management relies on antigen avoidance and symptomatic treatment, as necessary. Many open research questions remain, however, including what factors other than tick bites may lead to the development of this condition and whether AG sensitization per se may cause CAD, and if so, what can or should be done to mitigate this complication. Further studies are needed to address many aspects of AGS.

## Notes

***Patient consent*.** Patient consent is not applicable as no patient-specific data were included in this review.
